# Seroprevalence and Transmission of Human Influenza A(H5N1) Virus before and after Virus Reassortment, Cambodia, 2006–2014

**DOI:** 10.3201/eid2302.161232

**Published:** 2017-02

**Authors:** Sowath Ly, Paul Horwood, Malen Chan, Sareth Rith, Sopheak Sorn, Kunthea Oeung, Kunthy Nguon, Siam Chan, Phalla Y, Amy Parry, Reiko Tsuyuoka, Sovann Ly, Beat Richner, Denis Laurent, Sirenda Vong, Philippe Dussart, Philippe Buchy, Arnaud Tarantola

**Affiliations:** Institut Pasteur du Cambodge, Phnom Penh, Cambodia (S. Ly, P. Horwood, M. Chan, P. Y, S. Rith, S. Sorn, K. Oeung K. Nguon, S. Chan, S. Vong, P. Dussart,, P. Buchy, A. Tarantola);; World Health Organization, Phnom Penh (A. Parry, R. Tsuyuoka);; Ministry of Health, Phnom Penh (S. Ly);; Kantha Bopha Children's Hospitals, Siem Reap and Phnom Penh, Cambodia (B. Richner, D. Laurent);; GSK Vaccines R&D, Singapore (P. Buchy)

**Keywords:** viruses, influenza, H5N1 virus, reassortment, highly pathogenic, avian influenza virus, Cambodia, seroprevalence, seroepidemiologic study, transmission, humans, poultry

## Abstract

Thirty-five human influenza A(H5N1) cases were reported in Cambodia during 2013–2014 after emergence of a clade 1.1.2 reassortant virus. We tested 881 villagers and found 2 cases of pauci- or asymptomatic infection. Seroprevalence after emergence of the reassortant strain (0.2%) was lower than the aggregate seroprevalence of 1.3% reported in earlier studies.

Human influenza A(H5N1) virus infections in Cambodia were first detected in January 2005. Twenty-one cases, including 19 (90.5%) deaths, were reported during 2005–2012. In January 2013, researchers at the Institut Pasteur du Cambodge (IPC) in Phnom Penh, Cambodia, identified a new influenza A(H5N1) clade 1.1.2 reassortant virus in humans exposed to poultry (*1*). A sudden surge of 26 new human cases was observed in 2013, of which 15 (57.9%) resulted in death. In the first quarter of 2014, a total of 9 confirmed cases were reported: 8 (including 4 deaths) were caused by the clade 1.1.2 reassortant virus and 1 by clade 2.3.2.1 virus. Of the 8 clade 1.1.2 H5N1 cases, 3 occurred in villages in Kratie and Kompong Cham Provinces. We conducted studies in the 2 affected villages in these provinces in 2014 and compared our findings with those from 7 community seroprevalence studies our team conducted during 2005–2010**.**

## The Study

In the first week of February 2014, a suspected case and 2 laboratory-confirmed cases of the new influenza A(H5N1) clade 1.1.2 reassortant virus occurred in a village in Kratie Province (village 1; population 695); The cases were in 2 separate households. At the same time, a third confirmed case was identified in a village in Kompong Cham Province (village 2; population 921). The 3 patients with confirmed H5N1 virus infection were young children who had close contact with sick or dying poultry. Cases of H5N1 virus infection had also been reported in village 2 in April 2007 and December 2009.

We selected these 2 villages to conduct seroprevalence studies within a month of case occurrence to determine point-seroprevalence in the general population (Figure). Epidemiology teams from IPC and the Cambodia Ministry of Health interviewed and obtained serum samples from all village residents who provided informed consent. Sampling was repeated >2 weeks later to test for a possible increase in H5N1 virus antibody levels. We obtained National Ethics Committee for Human Research approval for all serostudies conducted as part of pandemic risk assessments.

**Figure:**
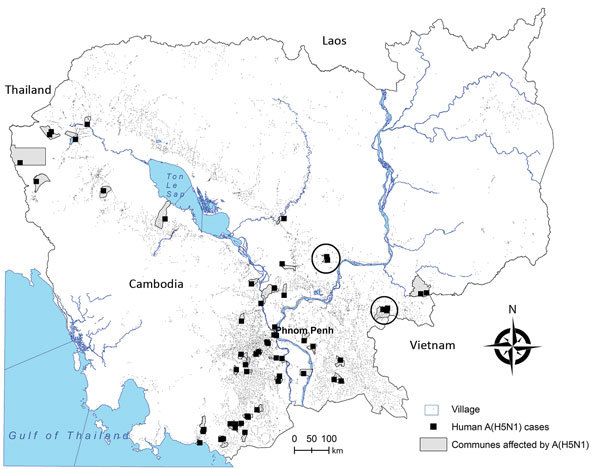
Geographic distribution of identified human cases in influenza A(H5N1)–affected villages, Cambodia, 2006–2014, Institut Pasteur du Cambodge, 2005–2014 (circles indicate areas investigated in 2014). Village distribution reflects population density. “Commune affected by A(H5N1)” refers to Cambodian communes in which A(H5N1) virus infection was laboratory-confirmed among humans or poultry.

We used hemagglutination inhibition (HI) and microneutralization assays as described previously (*2*) to test paired serum samples for H5N1 virus antibodies. Patients whose first serum sample was antibody-negative and whose second blood sample showed seroconversion (HI titer >80 and a microneutralization titer >40) were considered H5N1 virus positive (*3*). Patients who had detectable antibodies in the first serum sample and a >4-fold rise in antibody titer (minimum HI titer of 40 and microneutralization titer of 20) for the second sample were also considered H5N1 positive (*3*).

We used EpiData (EpiData Association, Odense, Denmark) to double-enter questionnaires. We excluded index cases from the analysis to assess risk of infection due to co-exposure or secondary transmission and calculated prevalence by using Poisson confidence intervals. We used the Fisher exact test to compare our data to those from community seroprevalence studies conducted before emergence of this H5N1 reassortant virus.

Paired samples were obtained from 238 (34.2%) of the 695 children and adults in village 1 and from 643 (69.8%) of 921 persons in village 2 (Figure). All persons in direct contact with the index case-patient in village 1 were screened, but 2 healthy family contacts of the second case-patient in village 1 refused participation. By laboratory testing, we found 1 additional case of pauci- or asymptomatic human H5N1 infection from each village, giving an overall seroprevalence of 0.2% (95% CI 0.1%–0.9%). The paucisymptomatic seropositive patient in village 1 was the child of a cousin of the index case-patient who lived <30 m away; the asymptomatic seropositive patient from village 2 was unrelated to the index case-patient but lived <50 m away from her house. Participants from both villages reported extensive poultry deaths, suggesting widespread sources of exposure to the virus.

Seroprevalence studies to document the incidence and clinical spectrum of H5N1 virus infections help evaluate the effects of emergence of a reassortant virus on virus transmission in affected communities (4). We therefore compared these 2014 results to the 10-year findings of 7 community seroprevalence studies (Table). These comparisons were undertaken by the same teams of IPC epidemiologists and virologists around confirmed cases in H5N1 epidemic foci in Cambodia with the cooperation and support of the Cambodian Ministry of Health. 

**Table T1:** Seroprevalence of influenza A(H5N1) virus in affected villages (excluding index cases), Cambodia, 2005–2014*

Reference	Country, population type	Year	Clade	Testing method	Village population	No. positive/no. tested	% Positive (95% CI)†
(*5*)	Cambodia, villagers	2005	1	MN, WB	1,146	0/351	0 (0–0.01)
(*6*)	Cambodia villagers	2006	1	MN	1,192	7/674	1.0 (0–2.2)
(*2*)	Cambodia villagers	2007	1.1.1	MN, HI	847	18/700	2.6 (0.2–4.1)
Unpub. data	Cambodia, villagers	2009	1.1.1	MN, HI	927	10/622	1.6 (0.9–3.0)
Unpub. data	Cambodia, villagers	2010	1.1.2	MN, HI	452	0/366	0 (0–0.01)
This study	Cambodia, villagers	2014	1.1.2R‡	MN, HI	695	1/238	0.4 (0.1–3.0)
This study	Cambodia, villagers	2014	1.1.2R‡	MN, HI	921	1/643	0.1 (0.0–1.1)
Cambodia, 2004–2010 studies					4,564	35/2,713	1.3 (0.9–1.8)
Cambodia, 2014					1,616	2/881	0.2 (0.1–0.9)
Cambodia, all studies 2004–2014					6180	37/3,594	1.0 (0.7–1.4)

*Positive results were determined by using World Health Organization criteria. HI, hemagglutination inhibition assay; MN, microneutralization assay; WB, Western blot.  †Poisson interval. An additional study conducted in 2008 in Cambodia focused on 394 soldiers (majority), support personnel, and their families in a confirmed H5N1 virus hotspot. No infections were found (prevalence 0%; 95% CI 0.0%–0.01%). The collective exposure was different from previous studies (soldiers had little or no exposure to poultry), so these data were not included in the table.  ‡Clade 1.1.2 reassortant strain with internal and matrix genes originating from clade 2.3.2.1.

Studies we conducted prior to the emergence of the reassortant strain, including 2 unpublished serosurveys, tested 2,713 persons, of whom 35 (1.3%) were positive for H5N1 antibodies (Table). Our surveys of the population surrounding cases of infection by the reassortant H5N1 virus in 2014 shows a seroprevalence of 0.2%, lower than the aggregate seroprevalence of 1.3% for 2005–2010. This difference is statistically significant (p = 0.004). [Table T1]

Even if the 2 healthy but untested contacts in village 1 had been found positive, the seroprevalence of H5N1 virus in humans would still be significantly lower following the emergence of the clade 1.1.2 reassortant virus when compared with historical IPC data (*2**,**5**,6*). Despite the 2013–2014 surge in cases, there is no evidence of increased H5N1 virus transmission to humans or altered clinical and epidemiologic characteristics associated with the emergence of this reassortant virus. A review of literature showed that with the exception of 3 studies in China (*7*–*9*), all community seroprevalence studies conducted around H5N1 cases outside Cambodia found 0% prevalence (references not listed).

## Conclusions

Routine biocontrol measures in Cambodia are poor, and poultry surveillance is lacking. Farmers either delay reporting and detection, or sell sick poultry, due in part, to lack of compensation from the government. This enables poultry outbreaks to spread. These factors could explain why prevalence in populations around confirmed cases was 10-fold higher in Cambodia than elsewhere until 2012. 

The drop in the number of positive cases in the 2014 community serosurveys raises the possibility that villagers’ knowledge of avian influenza and prevention may have improved after 2012, probably as a result of Cambodian authorities’ commitment to and international funding for community education campaigns. Recent studies, however, have shown a >2.5-fold increase in the circulation of avian H5N1 virus in live bird markets in Cambodia during 2013 compared with 2011 (*10*,*11*). The peak in confirmed human infections with H5N1 reassortant virus during 2013-2014 may therefore be linked to this increased circulation of H5N1 reassortant virus among poultry, but it may also reflect improved surveillance in the public health sector because our 2014 seroprevalence study does not suggest increased transmissibility of the virus to humans.

Our study has limitations. Although there were 9 human cases of infection with H5N1 virus reported in 2014, all followed by direct-contact tracing, only 2 spatiotemporal seroprevalence studies were conducted around H5N1 clusters and foci. There was also some minor loss to follow-up. Transmission risk estimates in villages with clusters might be influenced by the role of host genetics in susceptibility to infection (*12**,**13*). Our findings may therefore apply to genetically vulnerable persons and may have been overestimated, but they remain comparable to or lower than historical data in Cambodia. It is difficult to compare seroprevalence results between studies due to differences in methods and the lack of consensus on neutralizing antibody levels associated with pauci- and asymptomatic influenza H5N1 infections. 

In conclusion, our findings provide no evidence for increased human-to-human H5N1 virus transmission in Cambodia after emergence of the clade 1.1.2 reassortant virus. Direct-contact tracing is essential, but regular community-wide seroprevalence studies may not be required. However, each new epidemic phase or emergence of a new reassortant virus must be carefully investigated to address specific public health and research questions that can be answered only in an outbreak setting.
